# Magnetic coupling at rare earth ferromagnet/transition metal ferromagnet interfaces: A comprehensive study of Gd/Ni

**DOI:** 10.1038/srep30092

**Published:** 2016-07-22

**Authors:** T. D. C. Higgs, S. Bonetti, H. Ohldag, N. Banerjee, X. L. Wang, A. J. Rosenberg, Z. Cai, J. H. Zhao, K. A. Moler, J. W. A. Robinson

**Affiliations:** 1 Department of Materials Science and Metallurgy, University of Cambridge, CB3 0FS, Cambridge, United Kingdom; 2 Department of Physics, Stanford University, Stanford, CA 94305, USA; 3 SLAC National Accelerator Laboratory, California 94025, USA; 4 Department of Physics, Loughborough University, Loughborough, LE11 3TU, United Kingdom; 5 State Key Laboratory of Superlattices and Microstructures, Institute of Semiconductors, Chinese Academy of Sciences, Beijing 100083, China; 6 Stanford Institute for Materials and Energy Science, SLAC National Accelerator Laboratory, Menlo Park, California 94025, USA; 7 Department of Applied Physics, Stanford University, Stanford, California 94305, USA

## Abstract

Thin film magnetic heterostructures with competing interfacial coupling and Zeeman energy provide a fertile ground to study phase transition between different equilibrium states as a function of external magnetic field and temperature. A rare-earth (RE)/transition metal (TM) ferromagnetic multilayer is a classic example where the magnetic state is determined by a competition between the Zeeman energy and antiferromagnetic interfacial exchange coupling energy. Technologically, such structures offer the possibility to engineer the macroscopic magnetic response by tuning the microscopic interactions between the layers. We have performed an exhaustive study of nickel/gadolinium as a model system for understanding RE/TM multilayers using the element-specific measurement technique x-ray magnetic circular dichroism, and determined the full magnetic state diagrams as a function of temperature and magnetic layer thickness. We compare our results to a modified Stoner-Wohlfarth-based model and provide evidence of a thickness-dependent transition to a magnetic fan state which is critical in understanding magnetoresistance effects in RE/TM systems. The results provide important insight for spintronics and superconducting spintronics where engineering tunable magnetic inhomogeneity is key for certain applications.

Modified magnetic interaction at the interfaces between different ferromagnetic materials can be utilised to engineer materials with magnetic properties that are significantly different from the bulk. A classical example of such a system is a rare-earth/transition-metal (RE/TM) ferromagnetic multilayer since in the presence of an external magnetic field the layers couple parallel, but the antiferromagnetic coupling between the layers can lead to novel magnetic states and phase transitions between them. Ferrimagnetic alloys and multilayers (antiparallel alignment between layers) are of great interest fundamentally and from an applied point of view. Ferrimagnetic alloys were until very recently the only magnetic system that showed ultrafast magnetisation switching induced by a femtosecond laser pulse^1^,^2^,^3^,^4^, and engineered magnetic multilayers are key components of superconducting spin valves^5^,^6^,^7^,^8^.

The theoretical groundwork of RE/TM systems was established by Camley and Tilley^9^,^10^; they modelled RE/TM multilayers and found that due to the different Curie temperatures (*T*_*C*_) of the RE and TM layers several phases occur as the effects of temperature and applied magnetic field are incorporated. At temperatures far below the *T*_*C*_ of the RE, the RE aligns with the applied field while the TM aligns antiparallel to both the RE and the applied field. This is called the *RE*-aligned state. At higher temperatures, the magnetisation of the RE decreases and a second-order phase transition to the *TM*-aligned state occurs in which the TM aligns parallel to the applied field and the RE aligns antiparallel to both the TM and the applied field. This simple picture was later confirmed experimentally^11^,^12^; however, the existence of a twisted state at the interface between bulk Fe and a thin layer of Gd (five atomic layers) was also predicted by Camley for certain values of the applied field^13^, but this has not been conclusively demonstrated.

Due to the interface-driven nature of RE/TM systems it is difficult to measure the exact magnetic structures of the individual layers using standard measurement techniques such as vibrating sample magnetometry or magnetoresistance measurements^14^,^15^,^16^. Additionally, at low temperatures the magnetisation of a RE layer in a RE/TM bilayer will be far larger than that of the TM layer, meaning it can be extremely difficult to deconvolute the two signals. Therefore, an element specific measurement technique such as x-ray magnetic circular dichroism (XMCD) is essential for investigating RE/TM systems. The energy of the x-rays used can be tuned to the absorption edges of particular elements, meaning only the magnetic response of specific layers is measured at any one time. Previous studies using XMCD have mainly confirmed the predictions of Camley and Tilley^9^,^10^: Barth *et al*. observed evidence of the transition between the RE- and TM-aligned state as a function of temperature in a Ni/Gd bilayer (7.5 nm and 5 nm thick, respectively)^12^, while Koizumi *et al*. observed the same behaviour in a Gd/Fe (2 nm/2 nm)_50_ superlattice measured at 20 K and room temperature^17^.

In this study we present extensive XMCD results of Ni/Gd/Ni films allowing us to fully map the magnetic states as a function of applied field and temperature for a wide range of different Ni and Gd thicknesses (Fig. 1). Our results demonstrate the full richness of the magnetic state diagram of RE/TM systems and shed new light on previous work that interpreted results based on an incomplete picture of RE/TM behaviour^14^,^18^.

## Results

We measured 15 polycrystalline samples by XMCD, each with different layer thicknesses, throughout a range of temperatures between 6 K and room temperature. Since the magnetic signal that is detected by XMCD is the projection of the magnetic moment onto the wavevector of the x-ray, the magnet coils are placed such that the applied magnetic field is parallel to the propagation direction of the x-rays. The easy-axis of the thin films is in-plane, so for effective measurement of the magnetic moment of the samples as small an angle as possible is desirable between the plane of the samples and the x-ray propagation; here we used an angle of 30 degrees as the films are measured in transmission.

**Figure 1:**
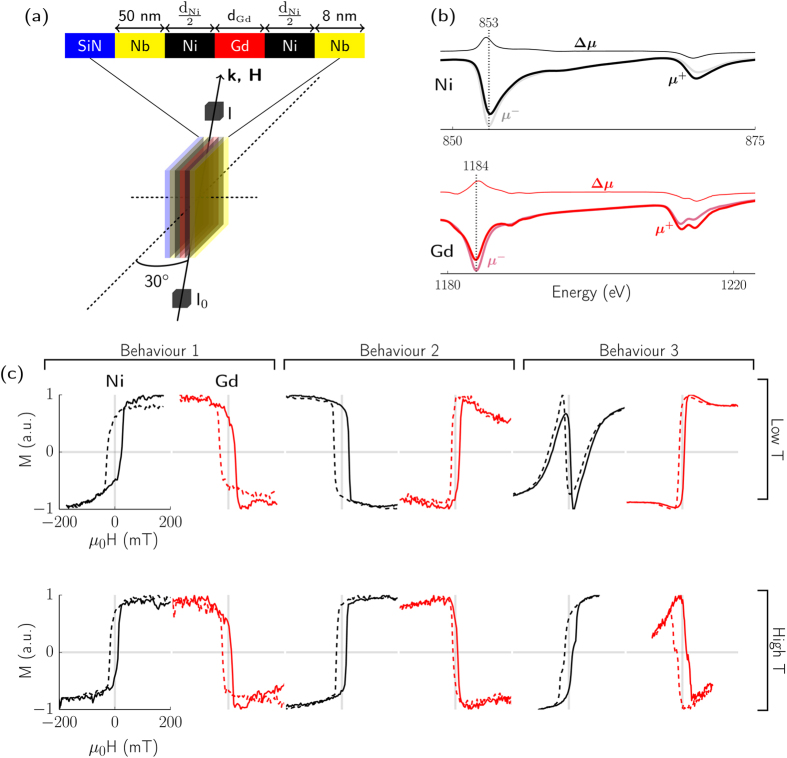
(**a**) Diagram of experimental setup showing the structure of the samples, the direction of the incident x-rays, and the position of the measuring photodiodes (*I*_0_ and *I*). (**b**) Examples of XMCD spectra taken at ~6 K for Ni (i) and Gd (ii). The XMCD was measured at the Ni *L*_3_ and Gd *M*_5_ absorption edges, respectively, marked by dotted lines. The two signals *μ*^+^ and *μ*^−^ are from positive and negative saturation, while Δ*μ* is the difference between the two, vertically offset for clarity. (**c**) Hysteresis loops measured between 5 and 10 K (low T), and 45 and 65 K (high T), that are representative of the three observed behaviours (N.B. Axis on all plots identical). The (total) Ni and Gd thicknesses of each of the samples are (from left to right): 5 nm Ni and 4.5 nm Gd, 6 nm Ni and 8 nm Gd, and 8 nm Ni and 6 nm Gd. The last sample is the same as that from which the XMCD spectra are shown in (**b**).

The measured samples displayed one of three behaviours as illustrated in Fig. 1(c) which shows element-specific magnetisation versus applied field (*M*(*H*)) hysteresis loops at high and low temperatures for three different samples that are characteristic of each behaviour. In the first behaviour the Ni layers follow the applied magnetic field (positive magnetisation in positive field) while the Gd layer remains antiparallel to the Ni and the magnetic field. This does not change as the temperature is increased. The hysteresis loops shown are from a sample with total Ni thickness of 5 nm, and Gd thickness of 4.5 nm (Note that the multilayers are of the form Ni/Gd/Ni, with equal Ni thicknesses either side of the Gd. This means that the thicknesses of the individual Ni layers will be half that of the total Ni thickness, and it is the total Ni thickness which is measured and stated in the text and figure captions.) In the second behaviour, the opposite is observed at lower temperatures: the Ni is antiparallel to the field while the Gd is parallel. Above a transition temperature, this state is reversed to the same as that in behaviour 1. The thicknesses of the Ni and Gd in this sample are 6 nm and 8 nm, respectively. The third behaviour is more complex: the Ni *M*(*H*) loops at low temperature displays a distinctive “N”-shape, indicative of significant magnetic inhomogeneity, in this case probably a fan-like state, in Ni during magnetisation reversal; the Gd layer undergoes a gradual transition over a temperature range of tens of kelvins from aligned parallel to the Ni to antiparallel. The sample displaying this behaviour in the figure had Ni and Gd thicknesses of 8 nm and 6 nm, respectively, and is the same sample as that for which the XMCD spectra are shown in Fig. 1(b).

## Model

**Figure 2:**
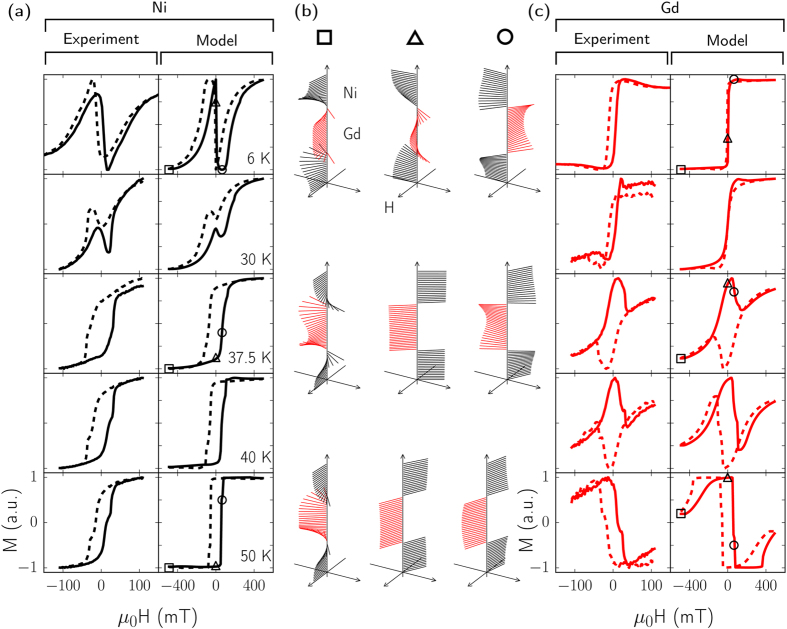
Comparison between the hysteresis loops measured by XMCD and those produced by the model, as well as detailed view of the micromagnetic structure of the sample at certain temperatures and field values extracted from the model. (**a,c**) Hysteresis loops, experimental and theoretical for Ni and Gd, respectively. The magnetisation has been normalised by the highest and lowest values of magnetisation within the loop to ease comparison. Shaped markers on some theoretical loops show the temperature and field values of the vector diagrams in (**b**). (**b**) Vector diagrams showing the direction of magnetisation of each atomic layer in the model system used to produce the hysteresis loops. The magnitude of the magnetisation is not shown. Black arrows represent Ni layers, red Gd, as labelled in the top left. The experimental hysteresis loops are from the same sample as that shown in Fig. 1(b,c) (behaviour 3), and has (total) Ni and Gd thicknesses of 8 nm and 6 nm, respectively.

A simple model exploring the competition between the different energies in the system allows us to confirm our explanation of the observed behaviour and illustrate possible micromagnetic structures within the samples which can reproduce the measured hysteresis loops. The model uses the Stoner-Wohlfarth framework by Mauri *et al*.^19^, and that of Camley and Tilley, wherein an iterative process is used to find the ground state of a stack of Ni and Gd atomic layers. We solve the following equation to find the angle of one magnetic moment at which the energy is a minimum:


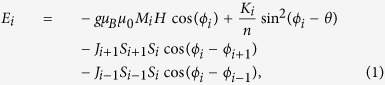


where *g* is the Landé g-factor, *μ*_*B*_ is the Bohr magneton, *μ*_0_ is the permeability of free space, *M* is the average magnetisation of the layer (in our case *M* is equal to the value of the magnetic moment of the atom for a perfectly ordered material), *ϕ* is the angle of a magnetic moment with respect to the applied magnetic field direction, *K* is the magnetocrystalline anisotropy energy, *θ* is the direction of the magnetic easy axis, *n* is the number density of the material of the layer, *J* is the exchange constant of the layer, and *S* is the magnitude of the magnetic moment of an atom in units of the Bohr magneton. We then iterate through the stack at each magnetic field value using the output of one iteration as the input for the next, until the difference between two iterations is less than a specified value. This process is repeated for each magnetic field value. Figure 2 shows a comparison between the results of the model and the temperature dependence of the third type of behaviour, which match very closely despite the simplicity of the model.

The model we use allows us to extract the micromagnetic structure from the system that produced the hysteresis loops since the modelled system is a one-dimensional stack of magnetic moments. The sum of the projections of the magnetic moments of each of the elements onto the direction of the applied field gives the total magnetisation at each field value, as shown in the hysteresis loops. The original angles of the each magnetic moment can also be plotted individually to give magnetic vector diagrams that show the inhomogeneity in the system at particular field values (Fig. 2(b)).

## Discussion

The first two observed behaviours can be understood in terms of the variation in the influence of the interfacial layers in the multilayer compared to that of the bulk. In the thinnest samples, the interfacial coupling will dominate and if the ratio between the Ni and Gd thicknesses favors the Ni, the Ni will control the behaviour of the Gd despite the higher magnetic moment of the Gd at lower temperatures (behaviour 1). For slightly thicker Gd layers, although the interfacial coupling still dominates the behaviour of the system, the Gd will dominate the Ni while its magnetic moment is not suppressed by the increasing temperature (behaviour 2).

We can now use the model to examine in detail the third observed behaviour. Magnetic vector diagrams produced by the model are shown in Fig. 2(b) and help illustrate our interpretation of the experimental results. They show the angles of the magnetisation for each atomic layer calculated by the model at a particular field value and temperature; the field values are indicated on the corresponding hysteresis loops. Figure 2(a,c) show the experimental and modelled hysteresis loops as a function of temperature. At 6 K and high fields, we expect the majority of moments in the both the Ni and Gd layers to be aligned parallel with the applied field direction. However, if the interfacial antiparallel coupling is maintained, there must be some moments canted away from this direction (seen in the vector diagrams marked with a square). Indeed, the model enforces strict antiparallel coupling at the interface, and the fact that the magnetisation at the maximum applied field is not the highest magnetisation measured in both the Ni and Gd loops supports this experimentally. The angle of the moments canting away from the applied field direction are highest at the interface, and due to the intra-layer exchange energies the angle changes continuously to allow the moments to align with the bulk further away from the interface. As the applied field is lowered the exchange energies dominate over the Zeeman energy and the change in angle of moments as a function of the distance from the interface is reduced (seen in the vector diagrams marked with a circle). This means that either the Ni or Gd are forced to align mostly antiparallel to the applied field due to the influence of the interfacial coupling. At low temperatures the Gd couples more strongly to the field and so forces the Ni to align antiparallel to both the Gd and the field. This is reflected in the hysteresis loops; as the field strength is decreased the magnetisation of the Ni decreases to negative saturation as the bulk Ni moments are forced by the intra-layer exchange energy to follow the Ni moments which are closest to the interface with the Gd. Then, as the field crosses zero, the Gd moments flip, following the field, and the Ni moments also all flip as shown by the sudden reversal in the hysteresis loop from negative to positive saturation (shown in the vector diagrams marked with a triangle).

As the temperature increases, the reversal of Ni becomes less pronounced due to a lowered influence of the Gd (Gd has a Curie temperature less than half that of Ni), and vanishes altogether by 50 K. Correspondingly, the Gd begins to display similar signs to those that the Ni displayed at low temperature; sudden magnetisation reversals that do not follow the direction of the applied field. This indicates that more of the Ni moments will be aligned with the field at high fields and higher temperatures, and we can see from the corresponding Gd loops that the Gd is for the most part completely under the influence of the Ni and remains antiparallel to both the Ni and the applied field for the majority of the applied field range. Any magnetic inhomogeneity that was present at the interface at lower temperatures has vanished for the most part.

The novel behaviour described above is reproducibly observed in multiple samples and so is a general behaviour. The measured samples were grown in four different growth runs, but we are confident that the observed effects are due to thickness dependence, and not stochastic variations in the growth conditions. This is because, firstly, samples from different growth runs display the same types of behaviours. Secondly, imaging the magnetic landscape on a micron-scale for three samples using a scanning SQUID microscope^20^, a sensitive probe of magnetic flux, shows consistent resolution-limited domain structure and uniform magnetisation strength for a variety of locations in each sample.

The measurement setup limits prevented us from applying a high enough field to observe true saturation. For example, we can see in the Ni loop at 6 K that the highest magnetisation in the loop is not the saturation magnetisation of the Ni at high fields. However, another factor influencing the saturation magnetisation is the measurement geometry; as previously mentioned, the magnetic field is necessarily applied at a 30 degree angle out of the plane of the samples.

**Figure 3:**
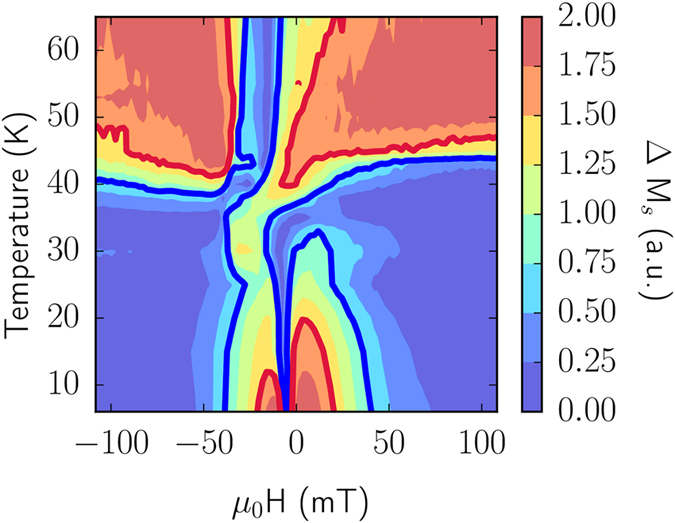
Phase diagram showing the degree of inhomogeneity present in an experimental multilayer as a function of applied field and temperature. The phase diagram is constructed from experimental hysteresis loops by taking the difference between the normalised magnetisation of Ni and Gd at each field value in a loop. This is then repeated for every temperature.

Figure 3 shows a magnetic state diagram for a particular sample that has been extracted from the experimental data as an illustration of how such a comprehensive study could help use the understanding gained about the RE/TM system to design new experiments and devices. To construct the figure, the Ni and Gd hysteresis loops are first normalized, so the maximum of the modulus of the difference is 2 (Δ*M*_*s*_ = 2); this corresponds to the Ni and Gd having oppositely saturated magnetisations, while Δ*M*_*s*_ = 0 corresponds to parallel Ni and Gd. We know that when the Ni and Gd layers are antiparallel there is no inhomogeneity at the interfaces, but the inhomogeneity will nucleate at the interfaces as the angle between the layers changes. Such a magnetic state diagram can also be constructed from the output of the model which could then be used to pick parameters that maximise magnetic inhomogeneity in devices in fields such as spintronics and superconducting spintronics where the manipulation of such magnetic structures is desirable.

In summary, we have performed a comprehensive experimental study of the RE/TM system using an extremely powerful measurement technique (XMCD). The use of the simple model allows a detailed view of the micromagnetic structure that is responsible for any of the three behaviours that were observed. The strict antiferromagnetic exchange coupling between RE and TM ferromagnets and its competition with the intralayer exchange energy and the Zeeman energy has been shown to lead to significant inhomogeneity in the system. The relative strengths of these energies, and thus the behaviour of the system can be tuned by varying the thickness of the individual layers within the multilayer, and our characterisation of the necessary thicknesses should enable the design of a new generation of devices that can utilise the magnetic inhomogeneity present at RE/TM interfaces including spintronics and superconducting spintronics. Our work will help to reinterpret the results of previous studies, and has consolidated our knowledge of another otherwise simple system that can be engineered to yield not only fundamentally interesting behaviours, but behaviours that have the potential to influence future technologies based on spintronics and superconducting spintronics^21^,^22^,^23^,^24^.

## Methods

### D.C. magnetron sputtering

The samples were grown at room temperature by d.c. magnetron sputtering in a system with a base pressure better than 10^−8^ mbar. The growth rate was 2.1 nm min^−1^ for Ni and 9.8 nm min^−1^ for Gd in argon at a pressure of 1.5 Pa.

### XMCD

XMCD was performed at beam line 13-1, SSRL at SLAC National Accelerator Laboratory. The geometry of the measurement setup is sketched in Fig. 1(a). Photodiodes measure the photon count before and after the x-rays pass through the sample. This allows a relative transmission to be measured while allowing for corrections to be made in fluctuations in the synchrotron beam. An electromagnet was mounted parallel to the x-ray propagation direction allowing applied fields in that direction of up to 250 mT, and a helium cryostat could cool the samples to approximately 6 K.

### Data Availability

The data supporting this paper is available open access at http://dx.doi.org/10.17863/CAM.609.

## Additional Information

**How to cite this article**: Higgs, T. D. C. *et al*. Magnetic coupling at rare earth ferromagnet/transition metal ferromagnet interfaces: A comprehensive study of Gd/Ni. *Sci. Rep*. **6**, 30092; doi: 10.1038/srep30092 (2016).
